# Comparison of clinically indicated replacement and routine replacement of peripheral intravenous catheters: A systematic review and meta-analysis of randomized controlled trials

**DOI:** 10.3389/fmed.2022.964096

**Published:** 2022-08-12

**Authors:** Ching-Yi Chen, Wang-Chun Chen, Jung-Yueh Chen, Chih-Cheng Lai, Yu-Feng Wei

**Affiliations:** ^1^Division of Pulmonary Medicine, Department of Internal Medicine, E-Da Hospital, I-Shou University, Kaohsiung, Taiwan; ^2^Department of Pharmacy, E-Da Hospital, I-Shou University, Kaohsiung, Taiwan; ^3^Institute of Biotechnology and Chemical Engineering, I-Shou University, Kaohsiung, Taiwan; ^4^School of Medicine, College of Medicine, I-Shou University, Kaohsiung, Taiwan; ^5^Department of Internal Medicine, E-Da Hospital, Kaohsiung, Taiwan; ^6^Division of Hospital Medicine, Department of Internal Medicine, Chi Mei Medical Center, Tainan, Taiwan; ^7^Department of Internal Medicine, E-Da Cancer Hospital, Kaohsiung, Taiwan; ^8^School of Medicine for International Students, College of Medicine, I-Shou University, Kaohsiung, Taiwan

**Keywords:** catheter-related infection, peripheral intravenous catheter, PIVC, phlebitis, routine replacement, clinically indicated replacement

## Abstract

**Background:**

It is unknown whether clinically indicated replacement of peripheral intravenous catheters (PIVCs) increases the risks of PIVC-associated complications and infections compared to routine replacement of PIVCs.

**Methods:**

We searched PubMed, the Web of Science, the Cochrane Library, Ovid MEDLINE, and Clinicaltrials.gov for randomized controlled trials (RCTs) that compare the safety outcomes of routine replacement and clinically indicated replacement of PIVCs were included for meta-analysis. The primary outcome was the incidence of phlebitis, and secondary outcomes included the risks of occlusion, local infection, infiltration, catheter-related bloodstream infection (CRBSI), and accidental removal of the PIVC.

**Results:**

A total of 9 RCTs involving 10 973 patients were included in this meta-analysis, of whom 5,546 and 5,527 were assigned to the study group (clinically indicated replacement of PIVCs) and control group (routine replacement of PIVCs every 72–96 h), respectively. The incidence of phlebitis in the study group was significantly higher than that in the control group [risk ratio (RR), 1.20; 95% confidence interval (CI), 1.01–1.44, *P* = 0.04, *I^2^* = 49%]. In addition, the study group was associated with a higher risk of occlusion (RR, 1.45; 95% CI, 1.08–1.95, *P* = 0.01, *I^2^* = 82%) and infiltration (fluid leaks) (RR, 1.27; 95% CI, 1.06–1.53, *P* = 0.01, *I^2^* = 72%) than the control group. However, no significant differences were observed in the risks of local infection (RR, 1.75; 95% CI, 0.38–8.16, *P* = 0.48, *I^2^* = 0%) and CRBSI (RR, 0.61; 95% CI, 0.08–4.68, *P* = 0.64, *I^2^* = 0%) between the study and control groups.

**Conclusion:**

The clinically indicated replacement of PIVCs may increase the risks of PIVC-associated phlebitis, infiltration, and occlusion compared to the routine replacement of PIVCs, but did not increase the risk of PIVC-associated infections. Based on these findings, routine replacement of PIVCs every 72–96 h maybe a preferred option than clinically indicated replacement of PIVCs.

**Systematic review registration:**

[www.crd.york.ac.uk/prospero/], identifier [CRD42022302021].

## Introduction

Peripheral intravenous catheter (PIVC) placement is one of the most common invasive procedures performed in acute care hospitals. More than 70% of hospitalized patients undergo placement of a PIVC to provide access for the intravenous administration of fluids, drugs, and nutrition (1–3). Although PIVCs can provide faster, less invasive and timely venous access for infusion therapy than other types of venous catheters, such as central venous catheters or peripherally inserted central catheters, they are occasionally associated with catheter failure and potential complications such as phlebitis, catheter dislodgement, occlusion, infiltration (fluid leakage), infusion site infection and catheter-related bloodstream infection (CRBSI) (3–6). Therefore, caring for and maintaining a PIVC to prevent these complications is an important issue.

According to the findings of several studies, the routine replacement of PIVCs to prevent intravascular catheter-related infections is recommended (7–9) and many hospitals have adopted this recommendation and routinely replace PIVCs. Nevertheless, several studies have demonstrated that replacing PIVCs only when clinically indicated, such as with the presence or signs of inflammation, infiltration, occlusion, infection, or blockage, was not associated with an increased risk of phlebitis or infections, but could reduce equipment costs, reduce staff workload, and improve patient comfort (10–14). Moreover, guidelines suggested that routine replacement PIVCs more frequently than every 72–96 h to reduce risk of infection and phlebitis in adults is not needed (15). A meta-analysis by Webster et al. reported no significant difference in the incidence rates of CRBSI, thrombophlebitis, all-cause bloodstream infection, mortality, and pain at the insertion site between clinically indicated and routine replacement of PIVCs (16). However, Buetti et al. recently conducted a large observational cohort study, and reported an association of increased risk of CRBSI when the catheters were replaced due to clinical indication instead of routine replacement every 96 h [incidence rate ratio (IRR) = 7.20; 95% confidence interval (CI), 3.65–14.22, *P* < 0.001], but no significant difference was observed in the reversion period (IRR = 1.35; 95% CI, 0.30–6.17, *P* = 0.69) (17). In 2021, three more randomized controlled trials (RCTs) reported this comparison in 2021 (9, 12, 13), however, the results were not consistent. To clarify this important issue after incorporating the updated information, we conducted this systematic review and meta-analysis to compare the safety outcomes of clinically indicated replacement and routine replacement of PIVCs.

## Methods

### Study search and selection

Comprehensive searches of PubMed, Embase, the Cochrane Library, and Clinicaltrials.gov for RCTs published before January 31, 2022 were performed. The following search terms were used: “catheter,” “vascular access device,” “catheterization,” “clinically indicated replacement,” and “routine replacement.” We only included RCTs that investigated the safety outcomes of clinically indicated or routine replacement of PIVCs. The inclusion criteria were: (1) clinically indicated replacement of PIVCs as the intervention group; (2) routine replacement of PIVCs every 72–96 h as the control group; (3) adult patients; (4) designed as a RCT; and (5) data regarding the clinical outcomes of interest were available. We excluded case reports, case series, observational studies, and retrospective cohort studies. Two investigators (CYC and WCC) independently screened and reviewed each study. In case of any disagreement, a third investigator (YFW) made the final decision. For each included study, we extracted the following data: publication year, study design, study site, and the incidence of complications. This study was conducted in accordance with the Preferred Reporting Items for Systematic Reviews and Meta-Analyses guidelines (18).

### Outcome measurements

The primary outcome was the incidence of phlebitis, and secondary outcomes included the risks of occlusion, local infection, infiltration, CRBSI, and accidental removal of the PIVC.

### Risk of bias assessments and data analysis

We used the Cochrane risk-of-bias tool (19) to assess the quality of the included RCTs, which was performed independently by two investigators (CYC and WCC). Any disagreement was resolved by consulting a third author (JYC). We performed all statistical analyses using Review Manager (version 5.3; Nordic Cochrane Center, Copenhagen, Denmark). Heterogeneity was evaluated using Q statistics generated by the χ^2^ test, and the *I*^2^ measure was used to assess statistical heterogeneity. Heterogeneity was defined as significant when *P* < 0.10 or *I*^2^ > 50%. We used a fixed-effects model when the data were homogeneous, and a random-effects model when the data were heterogeneous. We calculated pooled risks ratios (RRs) along with 95% CIs for outcome analyses.

## Results

### Study selection

**FIGURE 1:**
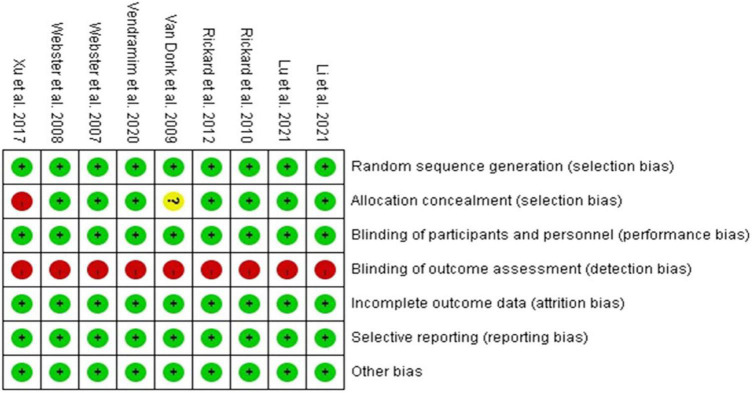
Algorithm for screening and identifying studies.

The search of the online databases yielded a total of 1,431 studies after excluding 229 duplicates. In addition, 1,372 studies were judged to be irrelevant after screening the titles, abstracts, and publications with no full text available. Furthermore, 50 studies were excluded after the full text of 59 articles was screened. Finally, 9 RCTs (9–11, 13, 14, 20–23) were included in this meta-analysis (Figure 1 and Supplementary Table 1).

### Study characteristics

Seven of the RCTs (9–11, 14, 21–23) were conducted at a single hospital, and 2 RCTs (13, 20) were multicenter studies (Table 1). Five studies (10, 20–23) were conducted in Australia, 3 were conducted in China (9, 13, 14), and 1 was conducted in Brazil (11). One study (21) included patients in a home program, and the other studies (9–11, 13, 14, 20, 22, 23) focused on hospitalized patients. In the control group, PIVCs were routinely replaced every 72 h in 5 studies (10, 14, 20, 22, 23), every 96 h in 3 studies (9, 11, 13), and every 72–96 h in 1 study (21). Overall, 10 973 patients were included in this meta-analysis, of whom 5,546 and 5,527 were randomly assigned to the study group (clinically indicated replacement of PIVCs) and control group (routine replacement of PIVCs every 72–96 h), respectively. Baseline characteristics of patients included in the enrolled studies were summarized in Supplementary Table 2.

**TABLE 1:** Characteristics of the included studies.

Study	Study design	Study site	Subjects	Timing of replacement		No. of patients	
					
				Intervention	Control group	Study group	Control group
Li et al. (13)	RCT	3 hospitals in China	Adult patients aged ≥ 18 years and expected use of PIVC > 4 days	Clinically indicated	Every 96 h	1,556	1,494
Lu et al. (9)	Single-blind, RCT	1 hospital in China	Adult patients aged ≥ 18 years, PIVC was used for the first time in the limb and had an expected use of > 4 days	Clinically indicated	Every 96 h	300	300
Rickard et al. (10)	Open-label parallel RCT	1 hospital in Australia	Adult patients requiring IV therapy ≥ 4 days in general medical or surgical wards	Clinically indicated	Every 72 h	185	177
Rickard et al. (20)	Open-label parallel RCT	3 hospitals in Australia	Adult patients had a PIVC *in situ* with expected duration ≥ 4 days	Clinically indicated	Every 72 h	1,593	1,690
Van Donk et al. (21)	RCT	Home program of 1 hospital in Australia	Adult patients who could be treated at home for an acute illness and had a 20-, 22-,or 24-gauge catheter inserted in an upper extremity	Clinically indicated	Every 72–96 h	105	95
Vendramim et al. (11)	Non-blinded, non-inferiority RCT	2 hospitals in Brazil	Aged at least 18 years, expected use of PIVC for at least 96 h, in select wards, intensive care units or surgical center	Clinically indicated	Every 96 h	672	647
Webster et al. (22)	RCT	1 hospital in Australia	Hospitalized adult patients expected to have a PIVC indwelling for at least 4 days	Clinically indicated	Every 72 h	103	103
Webster et al. (23)	RCT	1 hospital in Australia	Hospitalized adult patients expected to have a PIVC indwelling for at least 4 days	Clinically indicated	Every 72 h	379	376
Xu et al. (14)	Non-blinded cluster-RCT	1 hospital in China	Adult patients > 18 years of age who received catheter infusion; patients who were expected to use the indwelling catheter for ≥ 3 days; patients who used PIVCs for the first time during hospitalization	Clinically indicated	Every 72 h	553	645

RCT, randomized controlled trial; PIVC, peripheral intravenous catheter.

**FIGURE 2:**
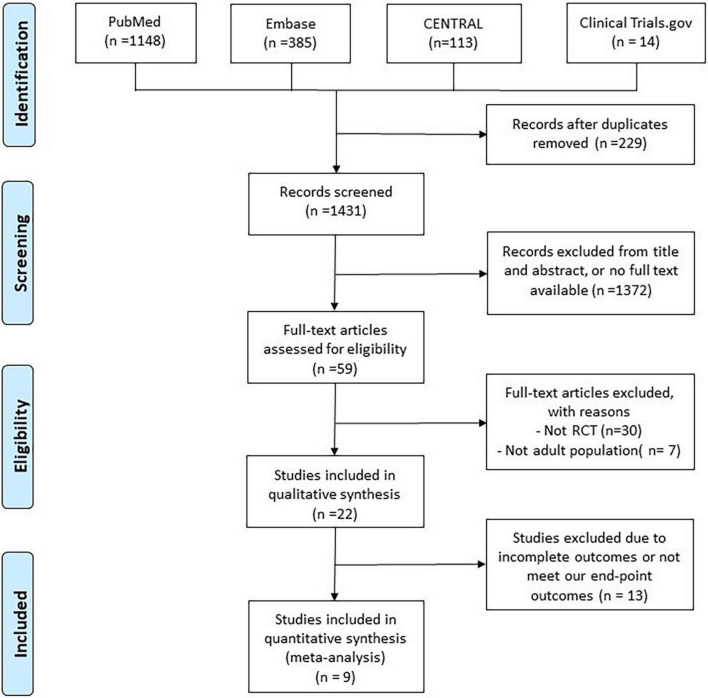
Risk of bias in each domain in each study.

Figure 2 illustrates the risk of bias in each study. The risk of bias was low in the categories of random sequence generation, complete outcome assessment, and selective reporting data in all included studies. For blinding, neither the participants nor clinical staff in any of the trials were masked due to the difficulty in clinical practice, but we still judged all of the trials to have a low risk of performance bias as the outcomes would not be affected by blinding. For allocation bias, 1 study was assessed to have a high risk, as randomization into 2 groups was performed by a research assistant according to a coin toss (14). Another study was assessed to have an unclear risk, as a detailed explanation of allocation concealment was not provided (21). With regards to outcomes, all trials were assessed to have a high risk of bias as the staff who assessed the outcomes (except for laboratory tests) were not blinded.

### Primary outcome

#### Phlebitis

**FIGURE 3:**
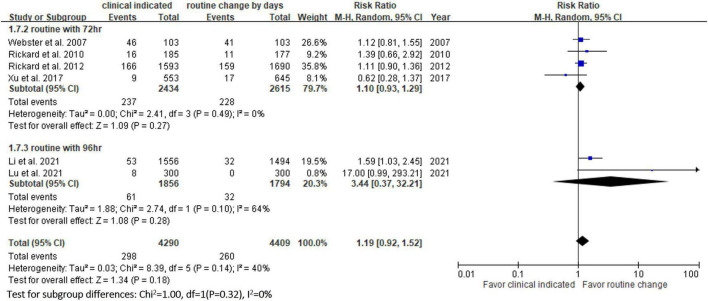
Forest plot of the risk of phlebitis.

In the pooled analysis of the 9 RCTs (9–11, 13, 14, 20–23), the incidence of phlebitis in the study group was 10.6% (579/5,446), which was significantly higher than that in the control group (9.0%, 498/5,527), with a RR of 1.18 (95% CI, 1.05–1.32, *P* = 0.04, *I^2^* = 49%) (Figure 3). The results remained unchanged in the random-effects model (RR, 1.21; 95% CI, 1.01–1.44). In subgroup analysis, according to the different schedule (every 72, 72–96, and 96 h) of routine replacement in the control group, the study group had a higher risk of phlebitis than the control group, however, the differences did not reach statistical significance (vs. every 72 h: RR, 1.12; 95% CI, 0.94–1.34, *P* = 0.20, *I^2^* = 0%; vs. within 72–96 h: RR, 1.29; 95% CI, 0.85–1.96; *P* = 0.24; vs. every 96 h: RR, 1.24; 95% CI, 0.79–1.93, *P* = 0.34, *I^2^* = 86%) (Figure 3).

#### Secondary outcomes

**FIGURE 4:**
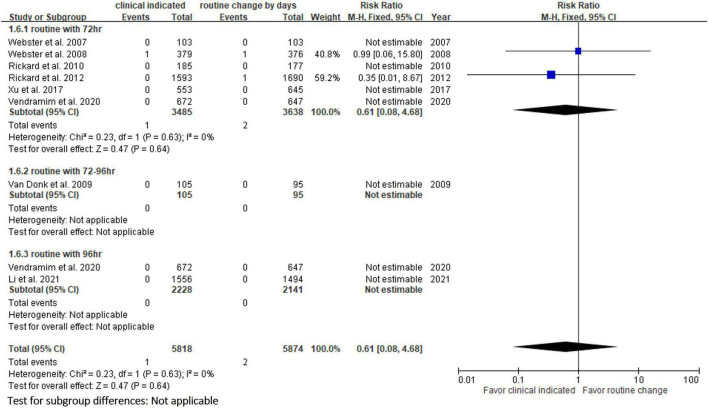
Forest plot of the risk of occlusion.

**FIGURE 5:**
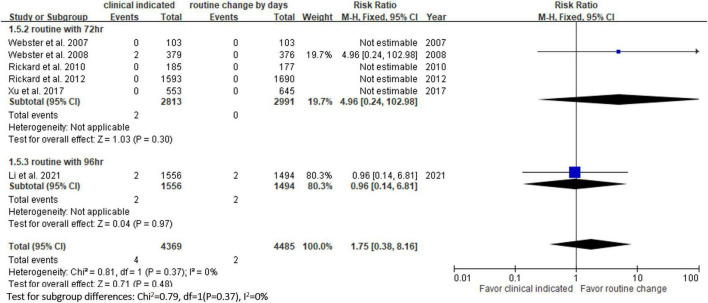
Forest plot of the risk of infiltration.

**FIGURE 6:**
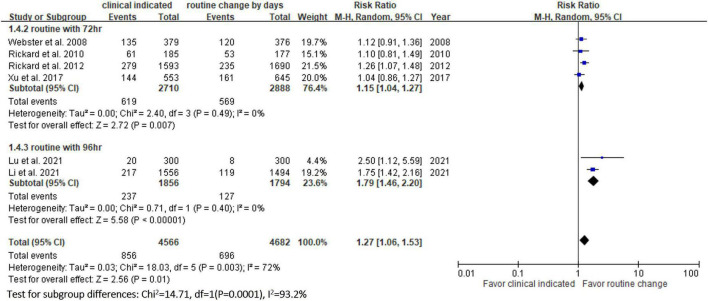
Forest plot of the risk of local infection.

**FIGURE 7:**
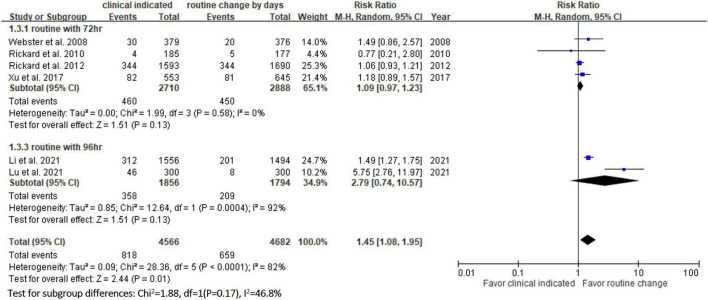
Forest plot of the risk of catheter-related bloodstream infection.

**FIGURE 8:**
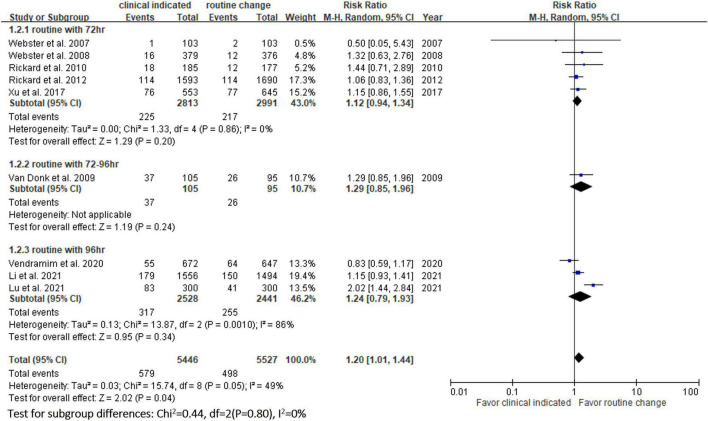
Forest plot of the risk of accidental removal.

Six RCTs (9, 10, 13, 14, 20, 23) reported the risk of occlusion, and the pooled analysis of these studies showed that the study group was associated with a significantly higher incidence of occlusion than the control group [18.0% (818/4,556) vs. 14.1% (659/4,682), RR, 1.45; 95% CI, 1.08–1.95, *P* = 0.01, *I^2^* = 82%] (Figure 4). These 6 studies (9, 10, 13, 14, 20, 23) also reported the risk of infiltration, and the study group was associated with a significantly higher risk of infiltration than the control group [18.8% (856/4,556) vs. 14.9% (696/4,682), RR, 1.27; 95% CI, 1.06–1.53, *P* = 0.01, *I^2^* = 72%] (Figure 5). Local infection was reported in 6 studies (10, 13, 14, 20, 22, 23), and no significant difference was observed between the study and control groups [0.09% (4/4,369) vs. 0.04% (2/4,485), RR, 1.75; 95% CI, 0.38–8.16, *P* = 0.48, *I^2^* = 0%] (Figure 6). In terms of CRBSIs, pooled analysis of 8 studies (10, 11, 13, 14, 20–23) showed that the study group had a lower risk of CRBSIs than the control group, but the difference did not reach statistical significance [0.02% (1/5,146) vs. 0.04% (2/5,227), RR, 0.61; 95% CI, 0.08–4.68, *P* = 0.64, *I^2^* = 0%] (Figure 7). Finally, no significant difference was found in the risk of accidental removal between the two groups [6.9% (298/4,290) vs. (5.9% 260/4,409), RR, 1.19; 95% CI, 0.92–1.52, *P* = 0.18, *I^2^* = 40%] (Figure 8) in the pooled analysis of 6 studies (9, 10, 13, 14, 20, 22).

Table 2 shows the results of subgroup analysis according to the schedule of routine replacement of PIVCs. Compared with routine replacement every 72 h or 96 h, clinically indicated replacement group had higher risk of infiltration (vs. 72 h: RR, 1.09; 95% CI, 1.04–1.27; vs. 96 h: RR, 1.79; 95% CI, 1.46–2.20). Otherwise, there was no significant difference between the study group and the control group (routine replacement every 72 h or 96 h) in terms of occlusion, local infection, CRBSI and accidental removal.

**TABLE 2:** Subgroup analysis according to the schedule of routine replacement of peripheral intravenous catheters.

Specific outcome	No. of studies	Rate ratio	95% CI
**Occlusion**			
vs. every 72 h	4	1.09	0.97–1.23
vs. every 96 h	2	2.79	0.74–10.57
**Infiltration**			
vs. every 72 h	1	1.15	1.04–1.27
vs. every 96 h	2	1.79	1.46–2.20
Local infection			
vs. every 72 h	5	4.96	0.24–102.98
vs. every 96 h	1	0.96	0.14–6.81
**Catheter-related blood stream infection**			
vs. every 72 h	6	0.61	0.08–4.68
**Accidental removal**			
vs. every 72 h	4	1.10	0.93–1.29
vs. every 96 h	2	3.44	0.37–32.21

## Discussion

In this meta-analysis, 9 RCTs (9–11, 13, 14, 20–23) involving 10 973 patients were included to compare the safety outcomes of clinically indicated replacement and routine replacement of PIVCs. Our findings show that routine replacement of PIVCs is superior to clinically indicated replacement, and this conclusion is supported by the following evidence. First, the overall risk of phlebitis in the pooled analysis of the 9 RCTs was significantly higher among the study group (clinically indicated PIVC replacement) than the control group (routine replacement of PIVCs every 72–96 h). A similar trend was observed in the subgroup analysis (72, 72–96, and 96 h in the control group), although the differences did not reach statistical significance. Second, the study group was associated with significantly higher risks of occlusion and infiltration than the control group. Further subgroup analysis also showed similar results.

Previous RCT study conducted by Lu et al. (9) who compared clinically indicated replacement with routine replacement every 96 h, and showed that the clinically indicated group had significantly higher risks of phlebitis (RR, 2.42; 95% CI, 1.60–3.66, *P* < 0.001), occlusion (RR, 6.61; 95% CI, 3.06–14.27, *P* < 0.001), infiltration (RR, 2.607; 95% CI 1.13–6.02, *P* = 0.020), and accidental dislodgement (RR, 2.03; 95% CI, 1.87–2.20, *P* = 0.013) (9). In addition, a previous meta-analysis conducted by Webster et al also reported similar results in terms of infiltration (RR, 1.16; 95% CI 1.06–1.26) and catheter occlusion (RR, 1.14; 95% CI 1.02–1.27, *P* = 0.002) in comparisons of a clinically indicated group and routine replacement group (16).

However, the incidence of phlebitis in our study was different from that in Webster’s meta-analysis (16), who found no significant difference in the incidence of phlebitis between clinically indicated and routine replacement groups (RR, 1.07; 95% CI 0.93–1.25, *P* = 0.34, *I^2^* = 0%). The difference between the present study and Webster’s study could be explained by the addition of two recent trials (9, 13) which were included in our updated meta-analysis. The RCT conducted by Li et al. reported that the incidence of phlebitis per patient was insignificantly higher in the clinically indicated group than in the routine replacement group [11.55% (171/1,489) vs. 10.3% (141/1,365), RR, 1.065; 95% CI, 0.937–1.212] (13). Another RCT conducted by Lu et al showed that the risk of phlebitis was higher in the clinically indicated replacement group than in the routine replacement group [27.7% (83/300) vs. 13.7% (41/300), RR, 2.416, 95% CI, 1.595–3.660] (9). After the addition of these two trials (9, 13) in our updated meta-analysis, the difference became significant after increasing the sample size.

The risk of PIVC-associated infections, including local infections and CRBSIs, was not different between the clinically indicated and routine replacement groups in the present study. These findings are consistent with a previous study (16), in which no difference was observed between clinically indicated and routine replacement groups in terms of local infection (2/2,260 vs. 0/2,346; RR, 4.96; 95% CI 0.24–102.98, *P* = 0.30) and CRBSI (1/3,590 vs. 2/3,733; RR: 0.61, 95% CI, 0.08–4.68, *P* = 0.64, *I*^2^ = 0%) based on the analysis of 7 trials involving 7,323 patients. These findings suggest that clinically indicated replacement does not increase the risk of catheter-associated infections compared to routine replacement.

This study has several limitations. First, several outcomes were analyzed based on data with heterogeneity, which may be due to various catheter devices, insertion sites, medical care, infusion medications, definitions of the complications, study facility, patients’ local (critical care or medical/surgery department), other lines placed at the same time, the demographic features of included patients (age, and disease severity). Second, the timing of routine replacement of PIVCs was not consistent, even though we performed subgroup analysis (72, 72–96, and 96 h) to minimize the time and measure differences (per patient, or per catheter), and selection bias could still exist between studies. Third, compared with routine replacement, clinically indicated replacement of PIVCs may reduce costs, prolong the indwelling time of PIVCs, reduce the workload of staff, and improve patient discomfort. As expected, we found that pooled analysis showed that the study group (clinically indicated replacement of PIVCs) had longer indwelling time per catheter than control group (routine replacement of PIVCs every 72–96 h) (mean difference: 21.17 h; 95% CI, 0.62–41.73, *P* = 0.04, *I*^2^ = 99%, Supplementary Figure 1). Only 3 earlier studies have reported cost as an outcome (20, 22, 23). We did not further assess these benefits of clinically indicated replacement due to unavailable or insufficient data in recent studies (9, 12, 13, 17). Therefore, further studies are still needed to clarify other outcomes such as cost-effectiveness.

## Conclusion

The results of this meta-analysis indicated that clinically indicated replacement of PIVCs was associated with increased risks of PIVC-associated phlebitis, infiltration, and occlusion compared to routine replacement. However, the risk of PIVC-associated infections, including local infections and CRBSIs, was not different between the two groups. Based on these findings, we suggest that routine replacement of PIVCs every 72–96 h maybe a preferred option than clinically indicated replacement in the clinical care of patients. Further large studies are still needed to verify these findings.

## Data availability statement

The original contributions presented in this study are included in the article/Supplementary material, further inquiries can be directed to the corresponding author/s.

## Author contributions

C-YC, W-CC, C-CL, and Y-FW: conceptualization and methodology. C-YC, W-CC, J-YC, and C-CL: software. C-CL and Y-FW: validation. C-YC and W-CC: formal analysis and writing—original draft preparation. J-YC and W-CC: investigation. C-YC, W-CC, and J-YC: resources and data curation. J-YC, C-CL, and Y-FW: supervision and writing—review and editing. C-YC, W-CC, J-YC, C-CL, and Y-FW: visualization. All authors contributed to the article and approved the submitted version.
