# Supplementary material for: Comparison of clinically indicated replacement and routine replacement of peripheral intravenous catheters: A systematic review and meta-analysis of randomized controlled trials
Source: Front Med (Lausanne). 2022 Aug 12;9:964096. doi: 10.3389/fmed.2022.964096 (PMC9411788; doi:10.3389/fmed.2022.964096)
Supplement: Supplementary file 1 [file Table_2.DOCX]

**Supplemental table S2. Baseline characteristics of included patients between the clinically indicated replacement group (study group) and the routine replacement group (control group)**

| Study | Mean age, year | | Male (%) | | Type of admission | | Number of Comorbidities (%) | |
| --- | --- | --- | --- | --- | --- | --- | --- | --- |
|  | Study group | Control group | Study group | Control group | Study group | Control group | Study group | Control group |
| Li et al, 2021 [13] | 58.6 | 59.1 | 59.1 | 58.4 | Medical: 51.7%  Surgical: 48.3% | Medical: 53.1%  Surgical: 46.9% | 0: 52%  1: 28%  ≥ 2: 20% | 0: 52%  1: 29%  ≥ 2: 19% |
| Lu et al, 2021 [9] | 56.3 | 58.7 | 65.3 | 66.3 | NA | NA | NA | NA |
| Rickard et al, 2010 [10] | 62.7 | 65.1 | 56 | 54 | Medical: 71%  Surgical: 29% | Medical: 70%  Surgical: 30% | 0: 5%  1: 11%  ≥ 2: 84% | 0: 3%  1: 12%  ≥ 2: 85% |
| Rickard et al, 2012 [20] | 55.1 | 55.0 | 64 | 61 | Medical: 18%  Surgical: 82% | Medical: 20%  Surgical: 80% | 0: 24%  1: 22%  ≥ 2: 54% | 0: 24%  1: 22%  ≥ 2: 54% |
| Van Donk et al. 2009 [21] | NA | NA | NA | NA | NA | NA | NA | NA |
| Vendramim et al. 2020 [11] | 59.7 | 59.9 | 50.4 | 49.1 | Medical: 88%  Surgical: 12% | Medical: 83%  Surgical: 17% | NA | NA |
| Webster et al. 2007 [22] | 60.2 | 63.1 | 48.5 | 47.6 | Medical: 45%  Surgical: 55% | Medical: 40%  Surgical: 60% | 0: 11%  1: 26%  ≥ 2: 63% | 0: 6%  1: 27%  ≥ 2: 67% |
| Webster et al. 2008 [23] | 60.1 | 58.8 | 65 | 62 | Medical: 63%  Surgical: 37% | Medical: 57%  Surgical: 43% | 0: 26%  ≥ 1: 74% | 0: 28%  ≥ 1: 72% |
| Xu et al. 2017 [14] | 58.7 | 56.2 | 58.8 | 56.7 | Medical: 53%  Surgical: 47% | Medical: 48%  Surgical: 52% | NA | NA |

NA, not applicable
