# Supplementary material for: Comparison of clinically indicated replacement and routine replacement of peripheral intravenous catheters: A systematic review and meta-analysis of randomized controlled trials
Source: Front Med (Lausanne). 2022 Aug 12;9:964096. doi: 10.3389/fmed.2022.964096 (PMC9411788; doi:10.3389/fmed.2022.964096)
Supplement: Supplementary file 2 [file Table_1.DOC]

**Supplementary Table S1**

**1.Pubmed**

| #1 | **Search: Catheter OR vascular access device OR catheterization**  "catheter s"[All Fields] OR "catheters"[MeSH Terms] OR "catheters"[All Fields] OR "catheter"[All Fields] OR ("vascular access devices"[MeSH Terms] OR ("vascular"[All Fields] AND "access"[All Fields] AND "devices"[All Fields]) OR "vascular access devices"[All Fields] OR ("vascular"[All Fields] AND "access"[All Fields] AND "device"[All Fields]) OR "vascular access device"[All Fields]) OR ("catheterisation"[All Fields] OR "catheterization"[MeSH Terms] OR "catheterization"[All Fields] OR "catheterisations"[All Fields] OR "catheterising"[All Fields] OR "catheterism"[All Fields] OR "catheterisms"[All Fields] OR "catheterise"[All Fields] OR "catheterised"[All Fields] OR "catheterizations"[All Fields] OR "catheterize"[All Fields] OR "catheterized"[All Fields] OR "catheterizing"[All Fields]) | 384018 |
| --- | --- | --- |
| #2 | **Search: clinically indicated replacement OR routine replacement**  (("ambulatory care facilities"[MeSH Terms] OR ("ambulatory"[All Fields] AND "care"[All Fields] AND "facilities"[All Fields]) OR "ambulatory care facilities"[All Fields] OR "clinic"[All Fields] OR "clinic s"[All Fields] OR "clinical"[All Fields] OR "clinically"[All Fields] OR "clinicals"[All Fields] OR "clinics"[All Fields]) AND ("indicate"[All Fields] OR "indicated"[All Fields] OR "indicates"[All Fields] OR "indicating"[All Fields] OR "indicative"[All Fields] OR "indicatives"[All Fields] OR "indicators and reagents"[Pharmacological Action] OR "indicators and reagents"[MeSH Terms] OR ("indicators"[All Fields] AND "reagents"[All Fields]) OR "indicators and reagents"[All Fields] OR "indicator"[All Fields] OR "indicators"[All Fields] OR "indice"[All Fields] OR "indices"[All Fields]) AND ("replace"[All Fields] OR "replaceable"[All Fields] OR "replaced"[All Fields] OR "replaces"[All Fields] OR "replacing"[All Fields] OR "replacment"[All Fields] OR "replantation"[MeSH Terms] OR "replantation"[All Fields] OR "replacement"[All Fields] OR "replacements"[All Fields])) OR (("routine"[All Fields] OR "routinely"[All Fields] OR "routines"[All Fields] OR "routinization"[All Fields] OR "routinize"[All Fields] OR "routinized"[All Fields] OR "routinizing"[All Fields]) AND ("replace"[All Fields] OR "replaceable"[All Fields] OR "replaced"[All Fields] OR "replaces"[All Fields] OR "replacing"[All Fields] OR "replacment"[All Fields] OR "replantation"[MeSH Terms] OR "replantation"[All Fields] OR "replacement"[All Fields] OR "replacements"[All Fields])) | 31009 |
| #3 | **Search: ( Catheter OR vascular access device OR catheterization) AND (clinically indicated replacement OR routine replacement)**  ("catheter s"[All Fields] OR "catheters"[MeSH Terms] OR "catheters"[All Fields] OR "catheter"[All Fields] OR ("vascular access devices"[MeSH Terms] OR ("vascular"[All Fields] AND "access"[All Fields] AND "devices"[All Fields]) OR "vascular access devices"[All Fields] OR ("vascular"[All Fields] AND "access"[All Fields] AND "device"[All Fields]) OR "vascular access device"[All Fields]) OR ("catheterisation"[All Fields] OR "catheterization"[MeSH Terms] OR "catheterization"[All Fields] OR "catheterisations"[All Fields] OR "catheterising"[All Fields] OR "catheterism"[All Fields] OR "catheterisms"[All Fields] OR "catheterise"[All Fields] OR "catheterised"[All Fields] OR "catheterizations"[All Fields] OR "catheterize"[All Fields] OR "catheterized"[All Fields] OR "catheterizing"[All Fields])) AND ((("ambulatory care facilities"[MeSH Terms] OR ("ambulatory"[All Fields] AND "care"[All Fields] AND "facilities"[All Fields]) OR "ambulatory care facilities"[All Fields] OR "clinic"[All Fields] OR "clinic s"[All Fields] OR "clinical"[All Fields] OR "clinically"[All Fields] OR "clinicals"[All Fields] OR "clinics"[All Fields]) AND ("indicate"[All Fields] OR "indicated"[All Fields] OR "indicates"[All Fields] OR "indicating"[All Fields] OR "indicative"[All Fields] OR "indicatives"[All Fields] OR "indicators and reagents"[Pharmacological Action] OR "indicators and reagents"[MeSH Terms] OR ("indicators"[All Fields] AND "reagents"[All Fields]) OR "indicators and reagents"[All Fields] OR "indicator"[All Fields] OR "indicators"[All Fields] OR "indice"[All Fields] OR "indices"[All Fields]) AND ("replace"[All Fields] OR "replaceable"[All Fields] OR "replaced"[All Fields] OR "replaces"[All Fields] OR "replacing"[All Fields] OR "replacment"[All Fields] OR "replantation"[MeSH Terms] OR "replantation"[All Fields] OR "replacement"[All Fields] OR "replacements"[All Fields])) OR (("routine"[All Fields] OR "routinely"[All Fields] OR "routines"[All Fields] OR "routinization"[All Fields] OR "routinize"[All Fields] OR "routinized"[All Fields] OR "routinizing"[All Fields]) AND ("replace"[All Fields] OR "replaceable"[All Fields] OR "replaced"[All Fields] OR "replaces"[All Fields] OR "replacing"[All Fields] OR "replacment"[All Fields] OR "replantation"[MeSH Terms] OR "replantation"[All Fields] OR "replacement"[All Fields] OR "replacements"[All Fields]))) | 1148 |

**2.EmBase**

| #1 | **Search: Catheter OR vascular access device OR catheterization** | 218660 |
| --- | --- | --- |
| #2 | **Search: clinically indicated replacement OR routine replacement** | 10142 |
| #3 | **Search: #1 AND #2** | 385 |

**3. CENTRAL**

| #1 | **Search: Trials matching Catheter OR vascular access device OR catheterization in Title Abstract Keyword - (Word variations have been searched)** | 32296 |
| --- | --- | --- |
| #2 | **Search: Trials matching clinically indicated replacement OR routine replacement in Title Abstract Keyword - (Word variations have been searched)** | 5098 |
| #3 | **Search: Trials matching " (Catheter OR vascular access device OR catheterization) AND (clinically indicated replacement OR routine replacement)"** | 113 |

**4. Clinical Trials.gov**

| #1 | **Search: Catheter OR vascular access device OR catheterization** | 2952 |
| --- | --- | --- |
| #2 | **Search:** **clinically indicated replacement OR routine replacement** | 10 |
| #3 | **Search: clinically indicated replacement OR routine replacement | Catheter OR vascular access device OR catheterization** | 14 |
