# Supplementary figures and images for: Comparison of clinically indicated replacement and routine replacement of peripheral intravenous catheters: A systematic review and meta-analysis of randomized controlled trials
Source: Front Med (Lausanne). 2022 Aug 12;9:964096. doi: 10.3389/fmed.2022.964096 (PMC9411788; doi:10.3389/fmed.2022.964096)

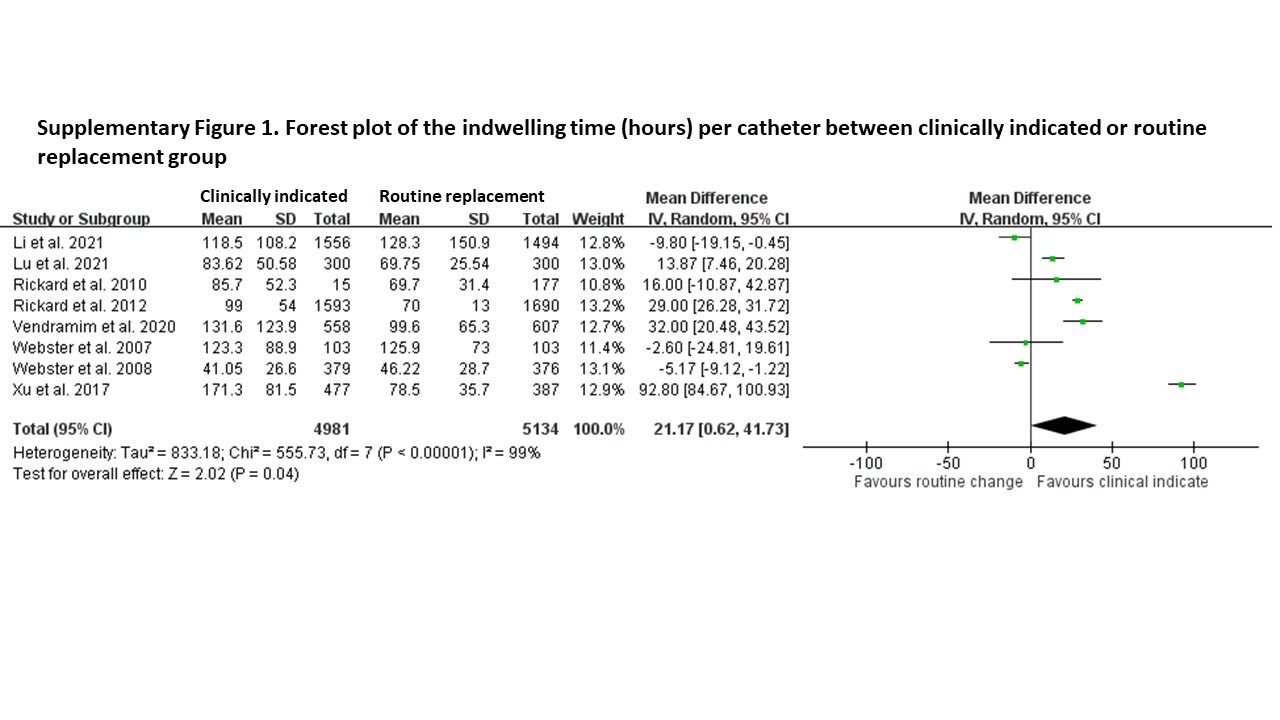

Supplement: Supplementary file 3 [file Image_1.jpg]
